# Predictive Value of Preoperative Morphology Parameters in Patients Undergoing On-Pump and Off-Pump Coronary Artery Bypass Surgery

**DOI:** 10.3390/jcdd11110375

**Published:** 2024-11-20

**Authors:** Krzysztof Greberski, Jakub Batko, Paweł Bugajski, Maciej Łuczak, Maciej Brzeziński, Krzysztof Bartuś

**Affiliations:** 1 Faculty of Health Sciences, University of Medical Sciences, 60-572 Poznan, Poland; 2 Department of Cardiac Surgery, J. Strus Municipal Hospital, 61-285 Poznan, Poland; 3 Department of Cardiovascular Surgery and Transplantology, Institute of Cardiology, Jagiellonian University Medical College, 30-688 Krakow, Poland; 4 Department of Cardiovascular Surgery, Medical University of Gdansk, 80-210 Gdansk, Poland

**Keywords:** CABG, OPCAB, cardiac surgery, morphology

## Abstract

Background: Coronary heart disease is the most common cause of death worldwide. It is responsible for almost a third of deaths in patients over the age of 35. Various biomarkers are currently being studied in detail for their value in predicting postoperative mortality in patients undergoing CABG. Aim: The aim of this study is to analyze the predictive value of certain blood morphological parameters in CABG and off-pump coronary artery bypass grafting (OPCAB). Methods: A total of 520 patients who underwent surgery in two consecutive years and underwent CABG (404) or OPCAB (116) were included in this retrospective study. Gender, age, comorbidities, five-year survival rate, detailed information on hospitalization, surgery, intensive care unit parameters and preoperative blood samples from the cubital vein were recorded. Inverse propensity treatment weighting was applied to adjust for confounding factors at baseline. Results: No differences were found between OPCAB and CABG as an isolated comparison. In the standardized population, patients with abnormal lymphocyte counts had an increased risk of death at one-year and five-year follow-up. In the standardized population, abnormal red blood cell distribution width (RDW-SD), neutrocyte-to-lymphocyte ratio (NLR), and platelet-to-lymphocyte ratio (PLR) were associated with increased mortality in each period analyzed. Conclusions: Abnormal PLR, RDW-SD and NLR are associated with increased early and late mortality in patients undergoing CABG and OPCAB. Abnormal lymphocytes are only associated with increased late mortality.

## 1. Introduction

Coronary heart disease is the most common cause of death worldwide. It is responsible for almost a third of deaths in patients over the age of 35 [1]. The risk factors for coronary heart disease include not only the aforementioned age, but also high blood pressure, dyslipidemia, diabetes mellitus, tobacco consumption, obesity and lack of exercise [2,3,4,5,6,7,8]. Coronary artery disease can lead to acute coronary syndrome, which is a life-threatening diagnosis [9]. It can be confirmed by characteristic symptoms, changes in the electrocardiogram and elevated high-sensitivity cardiac troponins [8]. In some cases, invasive and non-invasive imaging techniques can be helpful to confirm this diagnosis [8]. In case of a confirmed diagnosis, rapid percutaneous coronary intervention is crucial for a higher survival rate [8,10]. In patients with coronary artery anatomy that is unfavorable for percutaneous coronary intervention, emergency coronary artery bypass grafting (CABG) should be performed [7,8]. If the acute coronary syndrome leads to mechanical complications, CABG should be performed during such repair in patients requiring revascularization [7,8]. In chronic coronary artery disease, CABG is usually recommended to treat clinically significant narrowing of the main coronary arteries that cannot be treated with percutaneous coronary intervention [7]. Coronary artery bypass off-pump grafting (OPCAB), which does not require cardiopulmonary bypass, was developed as an evolution of the CABG technique [11]. It was thought to improve long-term outcomes, but several clinical trials have shown it to be superior only in terms of reducing short-term postoperative complications [12]. In addition, some reports suggest that it leads to less efficient myocardial revascularization [13].

Predicting postoperative mortality after CABG may be helpful in determining patients who are prone to this complication [14]. The most important predictors are New York Heart Association class above III, reoperation and preoperative dialysis [7,15,16]. Nowadays, many biomarkers that are readily available in daily practice are used to predict increased mortality risk in patients and in the general population [17,18,19,20,21,22,23]. The most common but underestimated biomarkers are neutrocyte-to-lymphocyte ratio (NLR), red blood cell distribution width standard deviation (RDW-SD), platelet-to-lymphocyte ratio (PLR), mean platelet volume (MPV) and platelet distribution width (PDW). All of these markers can be assessed by simple morphologic analysis of peripheral blood. However, few biomarkers have been extensively studied for their value in predicting postoperative mortality in patients undergoing CABG [24].

Therefore, the aim of this study is to analyze the predictive value of certain blood morphological parameters in coronary artery bypass grafting and to compare their value between CABG and OPCAB.

## 2. Materials and Methods

### 2.1. Study Population

**Figure 1:**
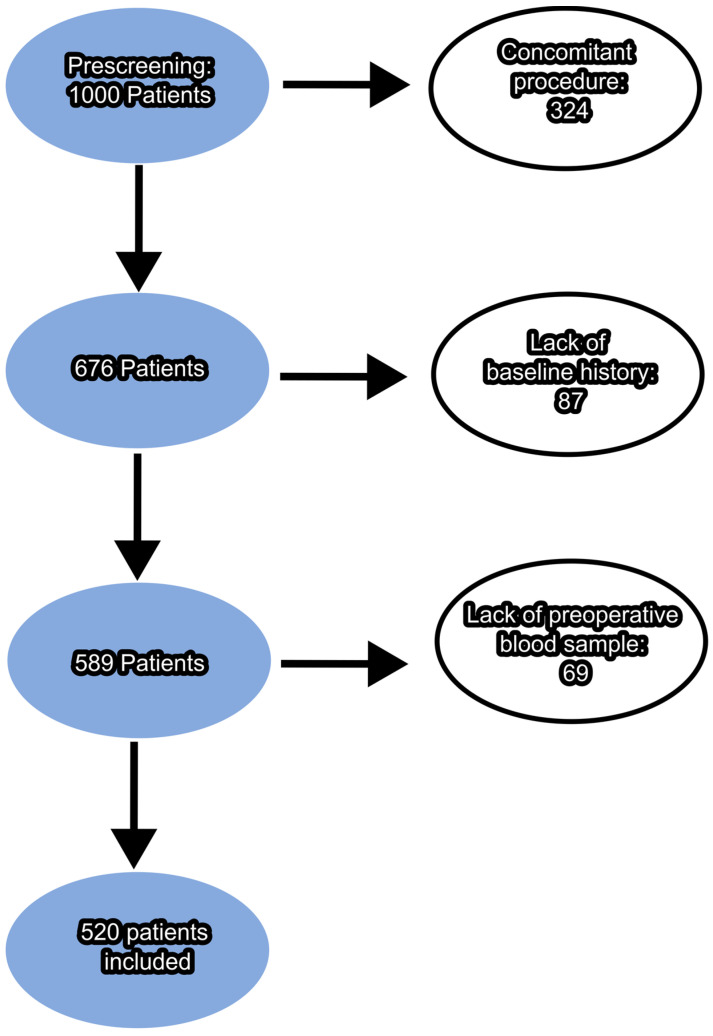
Flow chart for the inclusion of patients. The number of patients excluded based on each inclusion criterion is shown in white circles.

A total of 520 patients who underwent CABG (404) or OPCAB (116) in two consecutive years were included in this retrospective study. Gender, age, comorbidities, five-year survival rate, detailed information on hospitalization, surgery, intensive care unit (ICU) parameters and preoperative blood samples from the cubital vein were collected. Inclusion and exclusion criteria were as follows: patients who had undergone isolated CABG or OPCAB, had a blood sample taken preoperatively and had a complete medical history. All patients who did not have the above data or who underwent a concomitant procedure were excluded. A detailed flowchart of patient inclusion in the study can be found in Figure 1.

### 2.2. Blood Parameters

The following blood parameters were analyzed with the normal ranges and units given in Table 1: white blood cells (WBC), neutrocytes, lymphocytes, NLR, RDW-SD, platelets, PLR, MPV and PDW. For parameters for which there is no laboratory standard range, the standards proposed in experimental studies were used [25].

### 2.3. Statistical Analysis

The data were analyzed with IBM SPSS Statistics 29.0 (Predictive Solutions, Pittsburgh, PA, USA). The statistical size of the study population was calculated using the following parameters: Type I error rate equal to 0.05, Type 2 error rate equal to 0.2, proportion of subjects in each group equal to 0.2, proportion of individuals in the population exposed equal to 0.25, prevalence of the outcome in the population equal to 0.15, and estimated risk ratio equal to 2. The minimum sample size was calculated as 515. Categorical variables are presented as numbers (n) or percentages. Quantitative variables are presented as mean with standard deviation. The normal distribution was analyzed using the Shapiro–Wilk test. Differences between normally distributed quantitative parameters were assessed using the Student *t*-test. Differences between categorical variables were tested for independence using the chi-square test. Correlations were tested using the Pearson correlation (two-tailed test, α = 0.05, β = 0.2). Kaplan–Meier curves were generated to compare 5-year survival between patients operated on with different techniques and patients with blood parameters within and outside the range using the Mantel–Cox log-rank test. Odds ratios (OR) with 95% confidence intervals were calculated for 30-day mortality, one-year and five-year mortality and complications when comparing patients with blood parameters within and outside the standard range. Inverse propensity treatment weighting (IPTW) was used to standardize the baseline parameters in the population. A detailed description of the method can be found in our previous publication [26]. The population was standardized for: age, BMI, sex, CCS class, history of myocardial infarction, percutaneous coronary intervention, history of smoking, diabetes and its treatment method, hypertension, hyperlipidemia, chronic kidney damage, vascular disease, and left main coronary trunk obstruction. ORs with 95% confidence intervals (CI) for 30-day mortality, one-year and five-year mortality and complications were calculated for the standardized population, comparing patients with blood parameters within and outside the standard range. A *p*-value < 0.05 was considered statistically significant.

### 2.4. Bioethical Approval

The Bioethical Committee of University of Medical Sciences, Poznan, Poland approval was waived due to retrospective character of this study (Decision nr 748/24). The study protocol conforms to the ethical guidelines of the 1975 Declaration of Helsinki.

## 3. Results

### 3.1. Non-Standardized Population

#### 3.1.1. Baseline and Procedural Characteristics

The detailed characteristics of the study population are listed in Table 2. Statistically significant differences were found in the age of the patients, the duration of hospitalization and the duration of surgery.

#### 3.1.2. Preoperative Blood Parameters

The detailed results of the preoperative blood parameters are shown in Table 3. Significant differences were found both in the mean PDW and in the number of patients with a PDW outside the range between the OPCAB and CABG groups.

#### 3.1.3. Mortality

**Figure 2:**
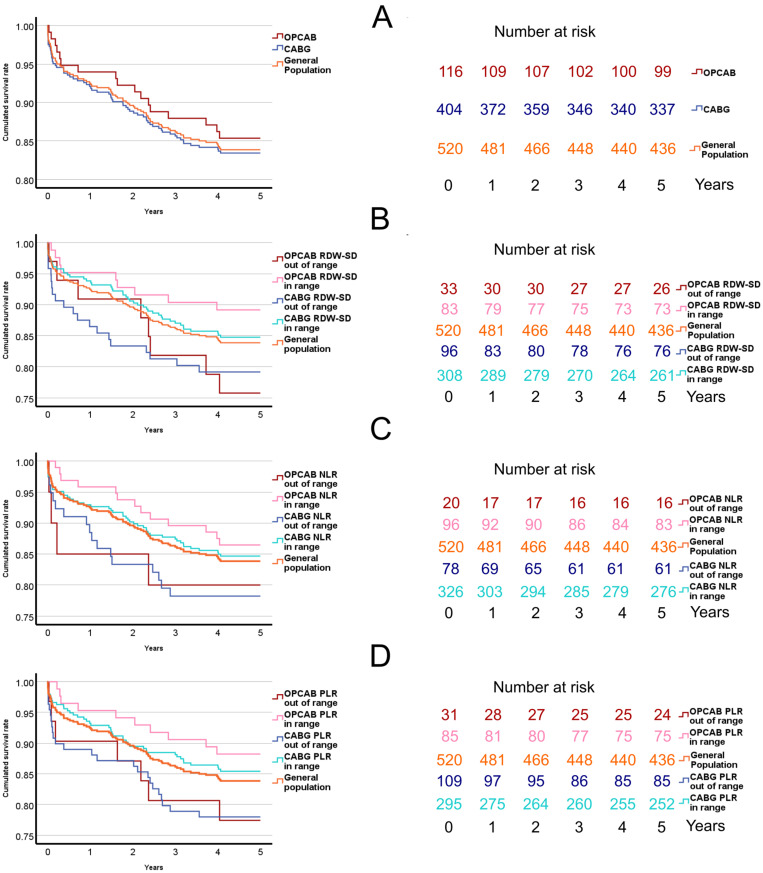
Survival curves of patients with blood parameters within and outside the normal range. The number of patients at risk is indicated for each survival curve. (**A**) Survival curve according to OPCAB, CABG and general population. (**B**) Survival curve of patients with RDW-SD within and outside the normal range over five years. (**C**) Patients with NLR within and outside the normal range of the five-year survival curve. (**D**) Patients with PLR within and outside the normal range of the five-year survival curve. CABG—coronary artery bypass graft, OPCAB—off-pump coronary artery bypass grafting, NLR—neutrocyte-to-lymphocyte ratio, RDW-SD—red blood cell distribution width standard deviation, PLR—platelet-to-lymphocyte ratio.

The overall mortality at hospitalization, after 30 days, after one year and after five years was 1.5%, 3.3%, 7.5% and 16.2%, respectively. Detailed survival curves comparing CABG and OP-CAB with Kaplan–Meier estimation can be found in Figure 2A. Detailed survival curves comparing patients with blood values within and outside the normal range can be found in Figure 2B–D. The OR of 30-day mortality in the general population was statistically significantly increased in the group of patients with PLR outside the normal range (3.195, 1.207–8.452 95% CI). The OR of 1-year mortality was statistically significantly increased in patients with RDW-SD outside the normal range (2.265, 1.157–4.436 95% CI). Both RDW-SD and PLR out of range increased the OR of 5-year mortality (1.658, 1.001–2.749 95% CI and 1.755, 1.071–2.874 95% CI, respectively). In the isolated CABG group, only the out-of-range platelet count increased the OR of one-year mortality (2.535, 1.026–6.262 95% CI).

#### 3.1.4. Correlations

In the general population, weak correlations were observed between Euroscore and lymphocytes (r = −0.097; *p* = 0.026), RDW-SD (r = 0.104; *p* = 0.018) and PLR (r = 0.121; *p* = 0.006). In the CABG group, a weak correlation was observed between operation time and RDW-SD (r = 0.136; *p* = 0.006); Euroscore and PLT (r = 0.105; *p* = 0.036) and PLR (r = 0.119; *p* = 0.017). In the OPCAB group, weak correlations were observed between 30-day and one-year survival and NLR (r = −0.187; *p* = 0.048 and r = −0.185; *p* = 0.049, respectively) and five-year survival and PLR (r = −0.218; *p* = 0.019).

### 3.2. IPTW Standardized Population

#### 3.2.1. Baseline and Procedural Characteristics

The detailed characteristics of the study population are listed in Table 4. Statistically significant differences were found in the duration of hospitalization and the duration of surgery.

#### 3.2.2. Preoperative Blood Parameters

The detailed results of the preoperative blood parameters are shown in Table 5. Significant differences were found both in the mean PDW, MPV, PLR and in the number of patients with PDW, MPV, WBC, Neutrocytes, Lymphocytes outside the range between the OPCAB and CABG standardized groups.

#### 3.2.3. Mortality

The all-cause hospitalization, 30-day, one-year, and five-year mortality rates were 0.9%, 2.8%, 6.3%, and 13.9%, respectively, in the standardized general cohort. The detailed OR of 30-day, 1-year and 5-year mortality in OPCAB, CABG and the general population in patients with each analyzed blood parameter within and outside of the standard range can be found in Table 6.

## 4. Discussion

### 4.1. Results Discussion

The aim of our study was to analyze the predictive value of daily blood morphology parameters for the survival rate of patients undergoing CABG, comparing two groups—classical CABG and OPCAB. When comparing the survival rate, we found no significant differences between these groups. Moreover, there was only one parameter—platelet count—whose abnormal values were exclusively associated with an increased risk of one-year mortality. Based on these results, it can be assumed that most blood parameters have a similar predictive value for CABG and OPCAB patients. Based on our results, a good predictor of 30-day mortality could be an abnormal PLR value, which is associated with an increased risk of death in CABG patients. On the other hand, a normal RDW-SD value proved to be a good predictor of long-term survival, both at one and five years. NLR out of range was associated with statistically significant differences in the overall survival curves of patients, but only in the 1-year period. Therefore, this result should be interpreted with caution. The correlations found were weak and should be analyzed with caution. Further statistically significant results were found when analyzing the comparison of the standardized populations. In general, patients with abnormal lymphocyte levels were found to have a more than 2-fold increased mortality rate [OR: 2.119 (1.227–3.661) 95%CI] at one-year follow-up compared to patients with lymphocytes within the standard range. This effect diminished at 5-year follow-up but remained statistically significant [OR: 1.580 (1.045–2.387) 95% CI]. In the standardized population, abnormal RDW-SD, NLR and PLR were associated with increased mortality in each time frame analyzed. PLR was associated with the greatest increase in mortality rate at 30-day and 5-year follow-up [OR: 6.149 (2.727–13.868) 95%CI and OR: 2.071 (1.427–3.008) 95%CI, respectively]. NLR was associated with the greatest increase in the probability of mortality at 1-year follow-up [OR: 2.836 (1.664–4.834) 95%CI].

Despite the different invasiveness of CABG and OPCAB, no significant differences were found between these groups in terms of short- and long-term mortality in the groups of patients with abnormal blood parameters in our study. This may indicate that even if the surgical technique affects the short-term homeostasis of patients, these differences are not as pronounced in terms of long-term mortality. We decided to additionally analyze the results of blood parameters based on their values within or outside the range to facilitate clinical implementation, as cut-off points are difficult to assess, remember and interpret in daily practice.

### 4.2. Historical Results Comparison

In a meta-analysis conducted by Pruc et al., abnormal preoperative PLR was associated with an increased risk of death in patients with acute coronary syndrome [26]. In the study by Tzikos et al., postoperative abnormal PLR values were associated with an increased risk of early mortality in cardiac surgery patients [27]. There were no previous publications on the long-term effects of preoperative PLR values on long-term mortality in CABG and OPCAB patients. Our results are consistent with previously published studies and demonstrate that PLR can be used as a predictive factor for premature mortality in patients with cardiovascular disease and patients undergoing cardiac surgery, particularly CABG.

Lee et al. analyzed the impact of RDW-SD changes on early adverse events after isolated CABG [28]. They demonstrated that RDW-SD changes are independent predictors of early adverse events after this procedure. Joshi et al. provided further insight into the importance of RDW-SD by analyzing its impact on predicting in-hospital mortality in patients without OPCAB and demonstrating that its abnormality is an isolated predictor of this complication [29]. Bujak et al. analyzed the crucial pathophysiology associated with the adverse effect of abnormal RDW-SD in patients with coronary artery disease and provided a molecular explanation for previous observations [30]. Gurbuz et al. investigated the impact of elevated RDW-SD on the long-term prevalence of cardiovascular events in CABG patients and found a strong association between elevated RDW-SD and a higher incidence of such events [31]. Our study proves that abnormal RDW-SD in patients undergoing CABG and OPCAB leads to an increased risk of both early and long-term mortality, further supporting previous findings.

Lim et al. demonstrated in his study on the impact of NLR on postoperative CABG outcomes that high preoperative NLR is associated with early mortality [32]. However, Sahin and Sisli, who analyzed OPCAB and CABG patients, found that only postoperative NLR abnormalities were associated with increased mortality [33]. Silberman et al. found NLR to be a predictor of operative and late mortality in a large cohort of patients [34]. Our study supports these findings.

### 4.3. What Are PLR, NLR and RDW-SD?

PLR is an inexpensive inflammatory biomarker that can be easily calculated in daily practice. It is calculated on the basis of the number of platelets and lymphocytes. It has long been known as a negative prognostic marker in oncology and hematology but is now also used in other areas of medicine. In the literature, it has been associated with complex endpoints, adverse cardiovascular events or atrial fibrillation in patients undergoing cardiac surgery [35]. Our study particularly highlights its greatest impact on the increased probability of death within the parameters analyzed.

The RDW-SD is an inexpensive, routinely measured parameter that is measured at every blood morphology examination. It measures the differences in erythrocyte size and volume. Its changes have been observed in various diseases, including peripheral arterial disease, heart failure and atrial fibrillation [36]. It has been suggested to be a predictor of complications and mortality in cardiac surgery [37,38,39]. Our study proves that RDW-SD is an additional predictor of postoperative mortality, with its abnormal values being slightly less associated with increased mortality compared to PLR and NLR

NLR is an inexpensive inflammatory biomarker that can be easily calculated in daily practice. It is calculated as a simple ratio of the number of neutrophils and lymphocytes. Recently, it has been analyzed in terms of evaluating the homeostasis of the immune system, as neutrophils are mainly responsible for the native immune response and lymphocytes for the adaptive immune response associated with antibody synthesis [40,41]. The NLR has previously been shown to be an independent prognostic factor for mortality in the general population and in various diseases [41,42,43,44,45]. Our study sheds light on its role as a predictive factor for mortality in patients undergoing CABG treatment.

### 4.4. Clinical Implications

Based on our results, patients with abnormal preoperative PLR should be monitored with greater caution during hospitalization and rehabilitation, as they are particularly prone to short-term mortality. Patients with abnormal NLR should be invited for frequent follow-up visits to perform further investigations and prevent premature death. Patients with abnormal preoperative NLR, PLR and RDW-SD are prone to increased postoperative short-term and long-term mortality.

### 4.5. Limitations and Further Studies

In our study, a retrospective analysis of 520 patients was performed. Only the preoperative blood parameters were analyzed. Our population was largely homogeneous except for age. To compensate for this confounder, we performed an IPTW analysis. Future studies should include the results of postoperative and long-term follow-up of blood parameters to determine the impact of their changes on patient mortality. In the future, other isolated interventions should also be included in the analysis to identify high-risk patients before the start of cardiac surgery. In addition, concomitant interventions that have been shown to reduce or increase the risk of bypass surgery should be included in the analysis [46,47]. Additional parameters, including lipoprotein levels, should be investigated as their levels have been shown in the past to be associated with a higher risk of death in patients with coronary syndromes [48]. The specific anatomy of the patient should be considered as another important factor in CABG [49,50,51]. In addition, cardiovascular imaging, especially advanced echocardiographic parameters, should be considered as an additional predictive factor [52,53,54].

## 5. Conclusions

Abnormal PLR, RDW-SD and NLR are associated with increased early and late mortality in patients undergoing CABG and OPCAB. Abnormal lymphocytes are only associated with increased late mortality.

## Figures and Tables

**Table 1:** Blood parameters with their units and normal ranges.

Blood Parameter	Unit	Laboratory/Experimental Normal Range
white blood cells	10^3^/μL	4–10
neutrocyes	10^3^/μL	1800–8000
lymphocytes	10^3^/μL	1500–4500
neutrocytes to lymphocytes ratio	-	0.43–2.75 male and 0.37–2.87 female
red cells distribution width standard deviation	fL	36–47
platelets	10^3^/μL	150–400
platelets to lymphocytes ratio	-	36.63–149.13 male and 43.36–172.68 female
mean platelet volume	fL	7.5–10.5
platelet distribution width	fL	6.1–11

**Table 2:** Baseline characteristics and details of hospitalization of analyzed study group. CABG—Coronary Artery Bypass Grafting, OPCAB—Off-Pump Coronary Artery Bypass Grafting, BMI—Body Mass Index, CCS—Canadian Cardiovascular Society, MI—Myocardial Infarction, PCI—Percutaneous Coronary Intervention, ICU—Intensive Care Unit.

		CABG (404)	OPCAB (116)	*p*
Age (years)		65.6 ± 7.4	68.1 ± 8.2	<0.001
BMI		29.1 ± 4.5	28.8 ± 4.1	0.558
Male		301 (74.5%)	90 (77.6%)	0.498
CCS Class	1	25 (6.2%)	8 (6.9%)	0.871
	2	257 (63.6%)	75 (64.7%)	
	3	120 (29.7%)	33 (28.4%)	
	4	2 (0.5%)	0 (0%)	
Previous MI		194 (48%)	62 (53.4%)	0.092
Previous PCI		18 (4.5%)	3 (2.6%)	0.367
Ever smoker	active	107 (26.5%)	30 (25.9%)	0.926
	previous	201 (49.8%)	60 (51.7%)	
Diabetes		183 (45.3%)	58 (50%)	0.371
Diabetes treatment	diet	33 (18%)	11 (19%)	0.977
	oral	88 (48.1%)	27 (46.6%)	
	insulin	62 (33.9%)	20 (34.5%)	
Hypertension		369 (91.3%)	106 (91.4%)	0.989
Hyperlipidemia		376 (93.1%)	110 (94.8%)	0.500
Chronic kidney injury		11 (2.7%)	6 (5.2%)	0.232
Vascular diseases	peripheral	66 (16.3%)	19 (16.4%)	0.885
	cerebral	50 (12.4%)	12 (10.3%)	
	both	69 (17.1%)	18 (15.5%)	
Left coronary artery main stem obstruction		24 (5.9%)	6 (5.2%)	0.754
Hospital stay (days)		12.7 ± 7.3	10.7 ± 2.7	0.001
Operation time (min)		173.4 ± 32.2	140.2 ± 30.1	<0.001
ICU time (hours)		31.2 ± 89.1	30.4 ± 67.1	0.253
Extubation time (hours)		17.5 ± 98.1	27.6 ± 117.3	0.510

**Table 3:** Detailed preoperative blood parameters. CABG—coronary artery bypass grafting, OPCAB—off-pump coronary artery bypass grafting, WBC—white blood cell count, NLR—neutrocyte-to-lymphocyte ratio, RDW-SD—red blood cell distribution width standard deviation, PLR—platelet-to-lymphocyte ratio, MPV—mean platelet volume, PDW—platelet distribution width.

Parameter		CABG (404)	OPCAB (116)	*p*
WBC	mean ± SD	7.9 ± 2.2	7.6 ± 2.8	0.177
	out of range (%)	60 (14.9%)	10 (8.6%)	0.083
Neutrocytes	mean ± SD	5 ± 1.7	4.7 ± 1.3	0.216
	out of range (%)	20 (5%)	1 (0.9%)	0.058
Lymphocytes	mean ± SD	2.1 ± 0.7	2.2 ± 2.5	0.52
	out of range (%)	93 (23%)	23 (19.8%)	0.528
NLR	mean ± SD	2.7 ± 1.6	2.6 ± 1.2	0.788
	out of range (%)	78 (19.3%)	20 (17.2%)	0.616
RDW-SD	mean ± SD	44.3 ± 4.7	45.1 ± 5	0.216
	out of range (%)	96 (23.8%)	33 (28.4%)	0.303
Platelets	mean ± SD	241 ± 71	229.6 ± 62.8	0.192
	out of range (%)	44 (10.9%)	13 (11.2%)	0.872
PLR	mean ± SD	130.8 ± 65.9	127.2 ± 62.7	0.56
	out of range (%)	109 (27%)	31 (26.7%)	0.956
MPV	mean ± SD	10.5 ± 1	10.3 ± 1	0.081
	out of range (%)	181 (44.8%)	43 (37.1%)	0.138
PDW	mean ± SD	13 ± 2.2	12.5 ± 2.3	0.007
	out of range (%)	318 (78.7%)	81 (69.8%)	0.046

**Table 4:** Baseline characteristics and details of hospitalization of standardized study group. CABG—coronary artery bypass grafting, OPCAB—off-pump coronary artery bypass grafting, BMI—body mass index, CCS—Canadian Cardiovascular Society, MI—myocardial infarction, PCI—percutaneous coronary intervention, ICU—intensive care unit.

		CABG (503)	OPCAB (507)	*p*
Age (years)		66.3 ± 7.5	66 ± 8.8	0.746
BMI		29.1 ± 4.4	29.2 ± 4	0.332
Male		376 (74.8%)	380 (75%)	0.942
CCS Class	1	30 (6%)	35 (6.9%)	0.911
	2	323 (64.2%)	311 (61.3%)	
	3	147 (29.2%)	161 (31.8%)	
	4	3 (0.6%)	0 (0%)	
Previous MI		237 (47.1%)	260 (51.3%)	0.186
Previous PCI		22 (4.4%)	13 (2.6%)	0.117
Ever smoker	active	132 (26.2%)	146 (28.8%)	0.560
	previous	253 (50.3%)	256 (50.5%)	
Diabetes		236 (46.9%)	231 (45.6%)	0.687
Diabetes treatment	diet	40 (8%)	41 (8.1%)	0.943
	oral	116 (23.1%)	110 (21.7%)	
	insulin	80 (15.9%)	80 (15.8%)	
Hypertension		458 (91.1%)	461 (90.9%)	0.943
Hyperlipidemy		471 (93.6%)	473 (93.3%)	0.825
Chronic kidney injury		15 (3%)	15 (3%)	0.982
Vascular diseases	peripheral	84 (16.7%)	90 (17.8%)	0.889
	cerebral	60 (11.9%)	55 (10.8%)	
	both	84 (16.7%)	90 (17.8%)	
Left coronary artery main stem obstruction		30 (6%)	24 (4.7%)	0.385
Hospital stay (days)		12.9 ± 7.1	10.6 ± 2.7	<0.001
Operation time (min)		179.8 ± 41.2	140.3 ± 31	<0.001
ICU time (hours)		32.3 ± 89.6	30 ± 63.3	0.091
Extubation time (hours)		17.9 ± 96.8	40.7 ± 149.7	0.111

**Table 5:** Detailed preoperative blood parameters. CABG—coronary artery bypass grafting, OPCAB—off-pump coronary artery bypass grafting, WBC—white blood white blood cell count, NLR—neutrocyte-to-lymphocyte ratio, RDW-SD—red blood cell distribution width standard deviation, PLR—platelet-to-lymphocyte ratio, MPV—mean platelet volume, PDW—platelet distribution width.

Parameter		CABG (503)	OPCAB (507)	*p*
WBC	mean ± SD	7.8 ± 2.1	7.8 ± 2.7	0.779
	out of range (%)	71 (14.1%)	47 (9.3%)	0.016
Neutrocytes	mean ± SD	5 ± 1.7	4.8 ± 1.4	0.555
	out of range (%)	23 (4.6%)	8 (1.5%)	0.006
Lymphocytes	mean ± SD	2 ± 0.7	2.2 ± 2.3	0.241
	out of range (%)	118 (23.5%)	80 (15.9%)	0.002
NLR	mean ± SD	2.8 ± 1.6	2.6 ± 1.2	0.293
	out of range (%)	96 (19.1%)	92 (18.2%)	0.712
RDW-SD	mean ± SD	44.4 ± 4.6	44.7 ± 4.9	0.484
	out of range (%)	117 (23.3%)	134 (26.5%)	0.244
Platelets	mean ± SD	239 ± 71	229.1 ± 65.3	0.054
	out of range (%)	57 (11.3%)	60 (11.9%)	0.803
PLR	mean ± SD	132.7 ± 68.8	122.9 ± 59.7	0.007
	out of range (%)	136 (27%)	123 (24.3%)	0.303
MPV	mean ± SD	10.4 ± 0.9	10.3 ± 0.9	0.010
	out of range (%)	225 (44.7%)	193 (38%)	0.032
PDW	mean ± SD	12.9 ± 2.2	12.4 ± 2.2	<0.001
	out of range (%)	393 (78.1%)	357 (70.5%)	0.006

**Table 6:** Odds ratio for 30-day, 1-year and 5-year mortality in patients with blood results within and outside of standard range. CABG—coronary artery bypass grafting, OPCAB—off-pump coronary artery bypass grafting, WBC—white blood cells, NLR—neutrocyte-to-lymphocyte ratio, RDW-SD—red blood cell distribution width standard deviation, PLR—platelet-to-lymphocyte ratio, MPV—mean platelet volume, PDW—platelet distribution width. Statistically significant results bolded. “-”—value cant be calculated.

		WBC Out of Range OR (95% CI)	Neutrocytes Out of Range OR (95% CI)	Lymphocytes Out of Range OR (95% CI)
OPCAB	30 days mortality	-	-	-
	1 year mortality	1.425 (0.409–4.969)	-	**3.425 (1.444–8.122)**
	5 years mortality	**2.243 (1.017–4.946)**	-	1.822 (0.927–3.581)
CABG	30 days mortality	0.657 (0.149–2.895)	**4.331 (1.166–16.082)**	1.533 (0.570–4.126)
	1 year mortality	1.261 (0.536–2.963)	2.735 (0.879–8.511)	1.637 (0.816–3.285)
	5 years mortality	1.225 (0.648–2.316)	1.734 (0.663–4.533)	1.330 (0.787–2.247)
General Population	30 days mortality	0.574 (0.134–2.448)	**4.067 (1.159–14.269)**	2.098 (0.928–4.743)
	1 year mortality	1.255 (0.603–2.611)	2.344 (0.792–6.933)	**2.119 (1.227–3.661)**
	5 years mortality	**1.689 (1.036–2.753)**	1.492 (0.601–3.703)	**1.580 (1.045–2.387)**
		Platelets out of range OR (95% CI)	MPV out of range OR (95% CI)	PDW out of range OR (95% CI)
OPCAB	30 days mortality	-	-	-
	1 year mortality	-	1.171 (0.510–2.691)	3.033 (0.891–10.326)
	5 years mortality	0.400 (0.121–1.323)	0.660 (0.357–1.220)	1.096 (0.584–2.055)
CABG	30 days mortality	**3.630 (1.336–9.862)**	1.735 (0.686–4.389)	0.595 (0.221–1.602)
	1 year mortality	2.112 (0.922–4.841)	1.328 (0.701–2.517)	0.952 (0.439–2.064)
	5 years mortality	1.691 (0.880–3.252)	1.298 (0.816–2.067)	0.736 (0.432–1.252)
General Population	30 days mortality	2.138 (0.848–5.386)	0.916 (0.425–1.977)	1.274 (0.511–3.178)
	1 year mortality	1.096 (0.509–2.360)	1.272 (0.766–2.114)	1.533 (0.805–2.920)
	5 years mortality	1.053 (0.609–1.821)	1.040 (0.724–1.493)	0.926 (0.619–1.384)
		RDW-SD out of range OR (95% CI)	NLR out of range OR (95% CI)	PLR out of range OR (95% CI)
OPCAB	30 days mortality	**4.767 (1.124–20.229)**	-	-
	1 year mortality	**2.477 (1.081–5.672)**	**4.189 (1.813–9.681)**	**3.342 (1.461–7.648)**
	5 years mortality	**2.958 (1.663–5.261)**	1.497 (0.767–2.924)	**2.396 (1.333–4.306)**
CABG	30 days mortality	2.525 (0.991–6.436)	2.015 (0.746–5.446)	**3.148 (1.251–7.925)**
	1 year mortality	**2.288 (1.176–4.449)**	**2.199 (1.089–4.441)**	1.900 (0.976–3.698)
	5 years mortality	1.448 (0.861–2.435)	**1.849 (1.083–3.159)**	**1.778 (1.089–2.903)**
General Population	30 days mortality	**3.139 (1.475–6.680)**	**5.358 (2.504–11.463)**	**6.149 (2.727–13.868)**
	1 year mortality	**2.349 (1.399–3.944)**	**2.836 (1.664–4.834)**	**2.390 (1.427–4.003)**
	5 years mortality	**1.896 (1.299–2.766)**	**1.678 (1.109–2.539)**	**2.071 (1.427–3.008)**

## Data Availability

Data are available from corresponding author upon reasonable request.
